# Graphene-Oxide-Enriched Biomaterials: A Focus on Osteo and Chondroinductive Properties and Immunomodulation

**DOI:** 10.3390/ma15062229

**Published:** 2022-03-17

**Authors:** Alessia Ricci, Amelia Cataldi, Susi Zara, Marialucia Gallorini

**Affiliations:** Department of Pharmacy, “G. d'Annunzio” University of Chieti-Pescara, Via dei Vestini 31, 66100 Chieti, Italy; alessia.ricci@unich.it (A.R.); cataldi@unich.it (A.C.); susi.zara@unich.it (S.Z.)

**Keywords:** graphene oxide, nanomaterials, osteogenesis, chondrogenesis, immunomodulation

## Abstract

Due to its exceptional physical properties, such as high electronic conductivity, good thermal stability, excellent mechanical strength, and chemical versatility, graphene has sparked a lot of interest in the scientific community for various applications. It has therefore been employed as an antibacterial agent, in photothermal therapy (PTT) and biosensors, in gene delivery systems, and in tissue engineering for regenerative purposes. Since it was first discovered in 1947, different graphene derivatives have been synthetized from pristine graphene. The most adaptable derivate is graphene oxide (GO). Owing to different functional groups, the amphiphilic structure of GO can interact with cells and exogenous or endogenous growth/differentiation factors, allowing cell adhesion, growth, and differentiation. When GO is used as a coating for scaffolds and nanomaterials, it has been found to enhance bone, chondrogenic, cardiac, neuronal, and skin regeneration. This review focuses on the applications of graphene-based materials, in particular GO, as a coating for scaffolds in bone and chondrogenic tissue engineering and summarizes the most recent findings. Moreover, novel developments on the immunomodulatory properties of GO are reported.

## 1. Introduction

Graphene, a two-dimensional monolayer sheet of sp^2^-hybridized carbon atoms bounded covalently to form a hexagonal network, has attracted tremendous attention and research interest owing to its exceptional physical properties, such as high electronic conductivity, good thermal stability, and excellent mechanical strength [1,2]. Discovered in 1947 [3], graphene and its derivatives have been explored for a wide range of applications, such as electronic and photonic devices, clean energy, and sensors [4].

There are two major fabrication methods related to graphene, defined as top-down and bottom-up procedures. The first method is based on exfoliation of the hexagonal 3D graphite reticulum of graphene to obtain 2D or 1D graphene layers. The second method uses the assembly of a single atom or molecules to accomplish 1D or 2D arrangements in graphene layers [5]. Liquid-phase exfoliation (LPE) of graphite is the best approach to obtain graphene in a large scale and at an economic price. This method is based on the capability of graphite to be dispersed in a specific organic solvent. By applying external forces, such as sonication or rapid agitation, van der Waals forces between graphene sheets break down, and a single purified monolayer can be obtained. This process can be even more improved by using surfactants [6]. Starting from pristine raw graphene, different derivatives have been synthetized, among which the most versatile are graphene oxide (GO), reduced graphene oxide (rGO), nanographenes (NGs), and graphene nanoribbons [7]. GO results from the chemical exfoliation and oxidation of layered crystalline graphite, while rGO is derived from the removal of oxygen groups from GO by reducing chemical agents, such as hydrazine, ascorbic acid, and many others, or through thermal or UV treatment of GO [8]. 

**Figure 1:**
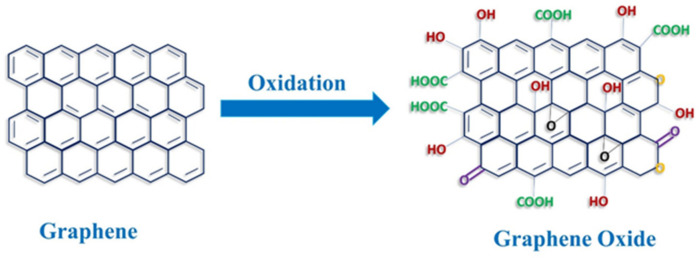
Chemical structure of graphene and graphene oxide.

GO presents different functional groups (Figure 1), such as hydroxyl and carboxylic groups in the edges and epoxy groups in the basal planes as well as phenol and lactone groups. Because of this structure, GO possesses better amphiphilic characteristics than pristine graphene. As a matter of fact, its hydroxylic and epoxy groups can establish hydrogen bonds, thereby making it dispersible in water or polar solvents. Moreover, its carbonic skeleton can interact with lipophilic groups [9]. The amphiphilic features of GO are essential for its biological usage. It has therefore broadly been reported that GO-coated materials support cell adhesion, growth, and differentiation [10]. Due to its attributes, GO has been reported to be capable of interfacing and co-operating with cells, growth factors, and hydrophilic substrates to improve the biocompatibility of several cell supports for regenerative purposes (Table 1) [11,12].

Over 10 years have passed since a single-layer graphene was exfoliated from highly oriented pyrolytic graphite [13], although research in this field is still broadly active. Thanks to its multiple properties and chemical versatility, there are several applications for graphene and its derivatives, and a plethora of research works have been made available in the last decade [14]. This review aims at summarize the main biomedical applications of GO-based materials with a particular focus on their osteoinductive and chondroinductive capabilities.

## 2. Biomedical Applications of GO

**Figure 2:**
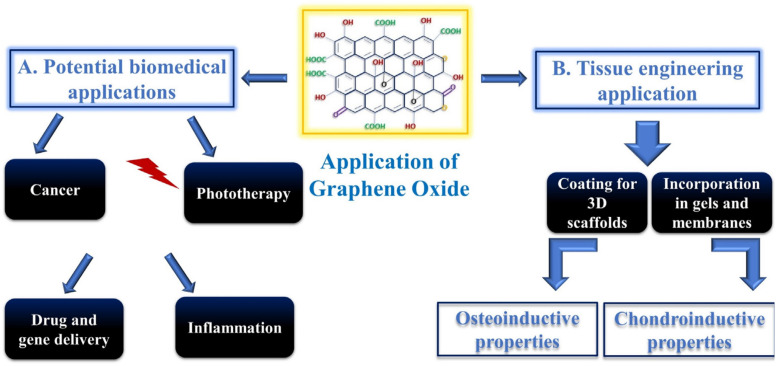
Biomedical applications of GO. (**A**) Among others, GO is used in phototherapy, cancer treatments, drug and gene delivery, and inflammation treatment. (**B**) Application of GO in tissue engineering and stem cell differentiation as osteoconductive and chondroinductive material [28].

Due to their exceptional properties, GO-based materials are nowadays investigated as biosensors [15], antibacterial agents [16], in photothermal therapy (PTT) [17], as gene delivery systems [18], and for applications in tissue engineering and regenerative medicine [19] (Figure 2A). Among all their potential functions, GO- and rGO-based scaffolds have particularly attracted attention because of their great clinical projection in tissue regeneration therapies, making them very promising candidates in this field (Table 1). Both GO and rGO have shown a strong impact on the proliferation and differentiation of implemented stem cells when applied on 3D scaffolds in bones, cardiac and neural regenerative medicine, skin and adipose tissues [20]. It has been reported that nano-GO incorporation in nanofibrous scaffolds, made of polycarbonate diol and isosorbide-based polyurethane, enhances the initial adhesion and spreading of myoblasts along with upregulation of myosin heavy chain mRNA levels. The ameliorated regenerative capacity has been ascribed to more suitable mechanical properties (flexibility) conferred to the material by GO enrichment [21]. In another study, autologous platelet-rich plasma (PRP) gels, containing various concentrations of GO, were prepared to promote tendon–bone interface healing and supraspinatus tendon reconstruction in a rabbit model. Again, the incorporation of GO improved the ultrastructure and mechanical properties of the PRP gels [22]. GO also enhances the characteristics of biomaterials used for skin wound dressing and wound healing. The addition of different percentages of GO (from 0 to 2%) on polyurethane material used for protecting skin wounds from external agents, such as microorganisms, and to improve its healing ameliorates its properties. As a matter of fact, polyurethane becomes more biocompatible in the presence of GO, and its antibacterial and mechanical properties appear to increase [23]. In another study, researchers highlighted the compatibility of GO–cellulose nanocomposites in the presence of endothelial cells (ECs) and their capability to improve in vitro EC migration and in vivo rat skin wound healing, thus inducing neo-vascularization and re-epithelization [24]. GO is also an applicable substrate in the presence of mesenchymal stromal cells from adipose tissue. It has been employed for enhancing peripheral nerve regeneration, demonstrating good biocompatibility and protection from the surrounding context, and furthermore improving nerve growth factor and glial-derived neurotrophic factor protein secretion [25].

The next sections will focus on the osteoconductive and chondroconductive properties of GO. Bone reconstruction represents a major challenge and is a global health problem. Enhancements in bone tissue engineering through the design of novel materials and coatings are continuously needed [26]. In parallel, cartilage growth and regeneration require a specific scaffold design that should be strong enough to stabilize the reconstructed cartilage until the newly synthesized ECM attains full mechanical stability and function. Furthermore, scaffold rigidity is important from a functional point of view, particularly in structures that enable nasal breathing, such as the septum, which is responsible for the shape and tension of its surrounding structures [27]. In contrast to the use of graphene in the osteogenesis process, which has been well known for a long time, there is little information available on the chondrogenic process in terms of support and differentiation, although studies on graphene and its derivatives have been increasing in recent years, as reported in the following paragraphs.

## 3. Osteoconductive Properties of Graphene

**Figure 3:**
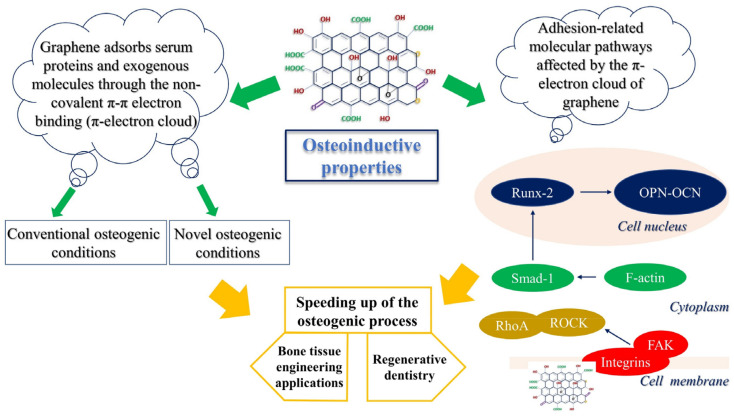
Osteoinductive properties of GO. Graphene enhances the osteogenic process by interacting with biomacromolecules through its π-electron cloud. In addition, GO is able to trigger cell-adhesion-related molecular pathways. FAK = focal adhesion kinase; SMAD1 = small mothers against decapentaplegic-1; ROCK = Rho-associated protein kinase; RhoA = Ras homologous GTPase; RUNX2 = runt-related transcription factor 2; OPN = osteopontin; OCN = osteocalcin.

Repairing major bone defects by means of bioactive materials, which can induce and support hard tissue formation, is the main goal of bone tissue engineering. Graphene and its derivatives, such as GO, offer outstanding osteoconductive features, making them the optimal choice for bone regeneration [29] (Figure 2B). Graphene and GO are used either alone or in combination with other biomaterials in the form of fillers in composites, coatings for both scaffolds and implants, or vehicles for the delivery of various signaling and therapeutic agents (Figure 3). The presence of graphene derivatives, even in small amounts, can significantly enhance mechanical strength, stiffness, and toughness of the material [30]. Regarding the optimal concentration of GO, there is no specific guideline to follow to obtain an ideal graphene support coating. However, the greatest improvements are usually observed at specific concentrations of graphene loading. Moreover, the presence of the functional groups in GO enables greater interactions with the matrix for more efficient load transfer [31]. In general, the best concentration for cell safety does not exceed 50 µg/mL for pristine graphene and GO and 60 µg/mL for rGO, and it should generally not exceed 1.5% *w*/*v* [32].

GO has been used as a reinforcing and osteoconductive agent in a variety of polymeric matrices, such as poly(vinyl alcohol) (PVA) scaffolds fabricated with laser sintering [33]; bioactive glass, which generally suffers from low fracture roughness [34]; cellulose suitable for scaffold production due to its high availability and renewability [35]; acrylic bone cements and titanium specimens, which exhibit lack of bioactivity and susceptibility to infection after implantation [19,36]; and hydroxyapatite-based scaffolds to improve surface roughness [37]. 

The ability of graphene to promote adherence and proliferation of mesenchymal stem cells (MSCs) and other various cell types, demonstrated for the first time by Kalbacova et al. [38], has been further documented in a plethora of literature. The capability of graphene derivatives to enhance cell features, such as adhesion and growth, resides in their strong proficiency to adsorb proteins, thereby creating a layer between cells and material surfaces. The π-electron cloud of graphene interacts with the inner hydrophobic core of serum proteins to form focal adhesion, an interaction that is even more fortified by GO with its oxygen functional groups [30]. Nayak and colleagues [39] were the first to demonstrate that graphene possesses osteoconductivity in MSCs under osteogenic conditions. From that moment, several studies have focused on this field with the aim of elucidating the molecular mechanisms underlying the osteoinductive properties of graphene and GO. The next section presents an overview of recent studies reporting the osteoconductive properties of GO, also focusing on the advantages of using new differentiation vehicles compared to traditional osteogenic media.

### 3.1. Enhanced Osteogenesis in the Presence of GO in Novel Differentiation Vehicles

Recent studies have focused on disclosing new potential differentiation strategies that could substitute the traditional ones (Figure 3) [40,41]. Osteogenic conditions are usually established in vitro by supplementing normal culture medium with a mixture of dexamethasone (Dex), ascorbic acid (Asc), and β-glycerophosphate (β-Gly) [42]. One proposed mechanism to explain how GO remarkably enhances osteogenesis is the strong ability of graphene to adsorb osteogenic supplements through noncovalent π–π electron binding, particularly when GO is present. By binding differentiation agents in the medium, GO creates a platform with a pool of concentrated substances, which enables and accelerates osteogenesis. Furthermore, the –OH group in GO has a particular affinity for Asc that is created by hydrogen bonding [43]. 

Several recent papers have reported the potential of GO in enhancing osteogenesis in traditional osteogenic conditions. Radunovic and coworkers [12] improved the biocompatibility of collagen membranes used for oral surgery of bone defects by coating them with GO. Furthermore, osteogenic differentiation and the anti-inflammatory response of pulp MSCs were enhanced. Many other recent works have reported on suitable osteoconductive characteristics of GO in traditional osteogenic conditions. For example, incorporating GO in a composite bone cement fabricated with polymethyl methacrylate (PMMA) enhanced the osteogenic differentiation of MSCs and progenitor cultures by increasing the number of voids and pores in the developed surface [44]. Kang and colleagues [45] constructed an MSC support made from a sheet of indium tin oxide coated with GO and gold nanoparticles, with the scaffold showing promising MSC osteogenic differentiation efficacy. Li et al. [46] incorporated GO into a methacrylated gelatin scaffold to enhance its properties for bone regeneration. The results showed that GO improved mineralization, and the mineralization was further increased when combined with silica-coated GO due to the enhanced biological activity of the BMP. rGO is also commonly used as a coating. Recently, Kang and coworkers [47] reported that a uniform coating of titanium surfaces with rGO allowed a significant increase of MSC proliferation after only 7 days and promoted the early expression of differentiation markers. The osteoconductive abilities of graphene derivatives have been confirmed not only in in vitro studies but also in vivo. As an example, collagen scaffolds are widely used as biomimetic scaffolds for bone implant [48], and their characteristics can be improved by GO. Zhou and colleagues [49] fabricated a collagen-based scaffold with apatite crystals and increasing concentrations of GO, which improved the coating efficacy. In this study, the authors first demonstrated that GO increased in vitro rat MSC adhesion and proliferation. Then, once implanted into the rat cranial defect, GO-coated scaffolds ameliorated the Ca/phosphate ratio, which was comparable to that of natural bones. In another study, Bahrami et al. [50] used collagen scaffolds (Col) coated with rGO (Col-rGO), showing promising in vitro results based on the capability of the scaffolds to enhance MSC viability and proliferation without cytotoxicity compared to scaffolds without rGO coating. Implantation of Col-rGO into rabbit cranial bone defects confirmed the pro-osteogenic ability of rGO after 12 weeks in vivo, with more bone formation being observed compared to noncoated Col support. In another recent study, GO was combined with poly-lactic-*co*-glycolic acid (PLGA), L-lysine, and gold nanoparticles. When implanted into rabbit radial defects, a synergistic effect was observed, with a high bone amount and mineralized collagen deposition [51].

In recent years, the possibility of investigating GO-dependent enhancement of osteogenic differentiation in novel osteogenic conditions has started to be a focus. As an example, based on the rationale that chondrocytes release substances that improve osteogenic differentiation during endochondral ossification, Kim et al. [52] compared MSC differentiation potential in traditional osteogenic differentiation medium and chondrocyte-conditioned medium in the presence of GO substrates. The results showed that GO substrates increased cell surface area and enhanced cell adhesion and osteogenic differentiation. Priming of cells with chondrocyte-conditioned medium could further induce synergistic osteogenesis on GO substrates. Another approach to promote skeletal tissue regeneration could be to enhance the release of ions, such as Ca^2+^, PO_4_^2−^, Li^+^, and Mg^+^ (the so-called inducerons), capable of starting stem cell differentiation from the surface of graphene derivatives [53]. In a recent work, it was reported that phosphate–GO-functionalized resorbable scaffolds used for bone regeneration were able to release inducerons in aqueous solutions, including Ca^2+^ and PO_4_^3−^. Calcium phosphate graphene could intrinsically induce osteogenesis in vitro and trigger ectopic bone formation in vivo in the presence of bone marrow stromal cells (BMSCs), thereby revealing phosphate–GO-functionalized materials as intrinsically inductive scaffolds capable of revolutionizing bone regeneration [54]. 

Given the widely demonstrated ability of GO to speed up the osteogenic process in both traditional and nontraditional conditions, the next section focuses on the most important MSC adhesion molecular pathways affected by graphene.

### 3.2. Stimulation of FAK-Related Pathways by GO Induces MSC Adherence and Osteogenic Differentiation

Several molecular studies on graphene-related osteoinductive capability have focused their attention on different pathways triggered in MSCs to enhance differentiation, such as phosphoinositide 3-kinases (PI3K)/protein kinase B (Akt)/glycogen synthase kinase (GSK)-3β/β-catenin [55], α5β1 and αvβ3 integrin [56], and reorganization of actin microfilaments [57]. As mentioned earlier, the π-electron cloud of graphene interacts with the inner hydrophobic core of serum proteins to form focal adhesions (FAs), an interaction that is even more enhanced by the oxygen group of GO [30]. FAs are specialized sites within the cell where clustered integrin receptors interact with the extracellular matrix (ECM) on the outside of cells and with the actin cytoskeleton on the inside, thus acting as transducers of mechanic stimuli, a process known as mechanotransduction [58]. It is broadly known that mechanical forces play a significant role in regulating the fate of MSCs, and it has been shown that controlling mechanical stimuli can in turn control MSC lineage specification [59], mainly in the presence of materials used as bone grafts [60]. It has been demonstrated that, in the presence of GO-enriched materials, such as polyethylene glycol (PEG)-based cryogel scaffolds, improved cell attachment and biocompatibility are mediated by FAK signaling activation [61]. Xie et al. [62] demonstrated that MSCs seeded onto graphene-coated polydimethylsiloxane (PDMS) substrates possess higher expression of integrin/FAK proteins and osteogenic markers compared to MSCs seeded onto PDMS alone (Figure 3). Again, FAK/p38 signaling pathways were proven to be involved in the enhanced osteogenic differentiation of MSCs in vitro, along with upregulated expression of focal adhesion (vinculin) on the GO-coated surface [63]. In another work, it was reported that MSC osteogenic differentiation induced by nano-GO modification on a titanium implant surface was driven by FAK/p38 signaling pathways, thus confirming that GO coating induced accelerated osteointegration and osteogenesis in vivo [63].

### 3.3. GO in Dentistry

GO has also aroused interest in the field of odontogenesis and regenerative dentistry (Table 1), as demonstrated by the plethora of works reporting the exceptional properties of graphene in this field [64]. 

Di Carlo and coresearchers [65] established a good and practical protocol to covalently coat cortical membranes commonly used in oral surgical dentistry with GO and analyzed the ability of this new material to promote adhesion, growth, and osteogenic differentiation of dental pulp stem cells (DPSCs) compared to membranes without coating. SEM analysis revealed that DPSCs formed a monolayer on discs, were contiguous to each other, and released granules of inorganic matrix, which was confirmed by alizarin red staining. Additionally, the cytotoxicity recorded in DPSCs grown on GO-coated discs was strongly reduced compared to cytotoxicity of DPSCs grown on titanium without GO. Moreover, GO augments the roughness of scaffold, and this characteristic improves cell attachment, proliferation, and differentiation. In another study, the same group [66] tested the ability of DPSCs to grow, proliferate, and differentiate onto GO foils in osteogenic conditions. The results showed that GO foils exhibited good biocompatibility and led to increased cell viability compared to polystyrene, which was used as a control. Furthermore, the authors reported that the cytotoxicity of GO foils was similar or lower compared to polystyrene, and earlier osteogenic differentiation was also enhanced with GO foils. Finally, the results showed the good chemical stability of GO sheets in aqueous solution for 7 days. 

Even though graphene-mediated MSC adhesion properties are essential for osteogenesis and odontogenesis, another crucial aspect to investigate is the immunomodulatory properties of graphene-based materials, which will be discussed in the next section.

## 4. Immunomodulatory Properties of GO in Osteogenic Conditions

The term osteoimmunology refers to the existing link between the immune and the skeletal systems. Both immune cells and cytokines contribute to the regulation of bone homeostasis, and bone cells also influence immune cell functions. Therefore, the immune microenvironment is crucial in determining the speed and outcome of bone healing, repair, and regeneration [67]. In the presence of a bone graft, an immune response is triggered independent of the material used, along with migration of immune cells in the affected area [68]. Currently, there are few studies on the influence of graphene on cells belonging to the immune system. The effects of GO coatings on immunoregulation and the subsequent impacts on osteogenesis are poorly understood. It has been shown that activated monocytes can communicate pro-osteogenic signals to MSCs, promoting osteogenesis [69]. Thus, nanomaterials specifically designed to provoke immune-mediated bone formation are still missing. It has been reported that conjugating the intrinsic immune characteristics of GO with the well-recognized osteoinductive capacity of calcium phosphate (CaP) in a biocompatible nanomaterial, namely maGO–CaP (monocytes activator GO complexed with CaP), may be a promising strategy to stimulate bone formation ex vivo and in vivo [70]. In another work, Su and coworkers [71] focused their attention on the effect of graphene on local immunity stimulation, particularly on the activity of macrophages, which are essential for osteoimmunology as they switch between the proinflammatory M1 phenotype or the anti-inflammatory M2 phenotype. GO-coated titanium (Ti–GO) surfaces exhibited good biocompatibility with the ability to stimulate the expression of osteogenic genes and ECM mineralization in human MSCs. Interestingly, macrophages seeded on Ti–GO showed proinflammatory activation under physiological conditions. SEM analysis revealed an M1 morphology, and a mild inflammation was also demonstrated by increased levels of proinflammatory interleukin (IL)-6, tumor necrosis factor-α (TNF-α), and IL-1β. When MSCs were seeded on Ti–GO in an environment mimicking acute inflammatory conditions, Ti–GO attenuated inflammatory responses, as shown by the downregulation of proinflammatory cytokines. Moreover, conditioned medium collected from macrophages stimulated by Ti–GO played a significant stimulatory role in hMSC osteogenic differentiation. This study confirms the strong potential of GO in immunomodulatory processes, even though further investigation about its influence on the immunity system is necessary. 

**Figure 4:**
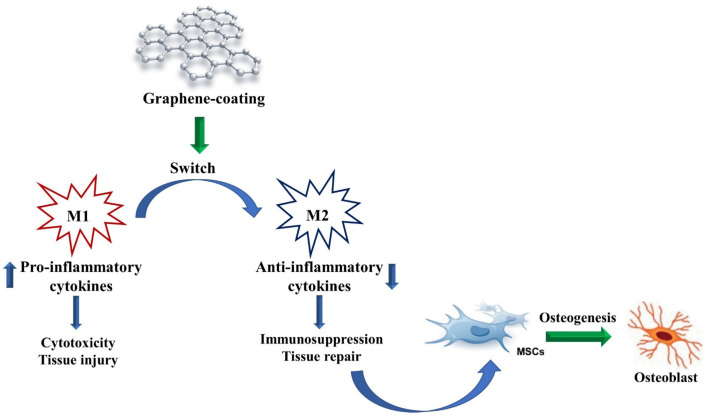
Effect of graphene on the immune system. Under the influence of graphene-coated materials, macrophages change their phenotype from the pro-inflammatory M1 to the anti-inflammatory M2 one, promoting tissue repair (osteogenesis). MSCs = mesenchymal stem cells.

Another main issue that can induce immunity system activation and lead to implant failure in the presence of bone grafts is the potential release of small debris from scaffolds used in standard orthopedic procedures. Chang and co-researchers [72] analyzed the osteolysis potential of graphene and preparation of GO particles in vitro in the presence of murine macrophages and in a murine model of calvaria. The results showed that GO induced an in vitro proinflammatory reaction characterized by increasing concentration of IL-6 and TNF-α released. However, the in vitro results were not confirmed in vivo. The authors speculated that the inflammatory reaction was a first step for inducing bone formation through activation of macrophages by graphene or GO, as previously described. As a matter of fact, macrophages switch from the proinflammatory M1 phenotype to the anti-inflammatory M2 phenotype in 72 h, thus promoting osteogenesis (Figure 4). Even though the available studies on the involvement of the immunity system in response to GO are promising, more in-depth research in this field is required.

## 5. Chondroinductive Properties of Graphene

Cartilage damaged by trauma has a limited capacity to heal. Thus, the regeneration of articular cartilage remains a major challenge in orthopedics and tissue engineering. Currently, treatment of small articular cartilage injuries involves administration of nonsteroidal anti-inflammatory drugs (NSAIDs), analgesics, corticosteroid, and hyaluronic acid (HA) injections, viscosupplementation, articular chondroplasty, and artificial joint replacement [73]. Larger defects are managed with osteochondral allograft or total joint arthroplasty. However, the future of managing cartilage defects lies in providing medication-free therapeutic solutions through cartilage regeneration using tissue-engineered cartilage [74]. The following paragraphs summarize the most recent works related to graphene and its derivatives, in particular GO, employed in chondrogenesis induction and cartilage regeneration (Table 1).

### 5.1. Graphene as a Substitute for Chondrogenic Differentiation Factors

**Figure 5:**
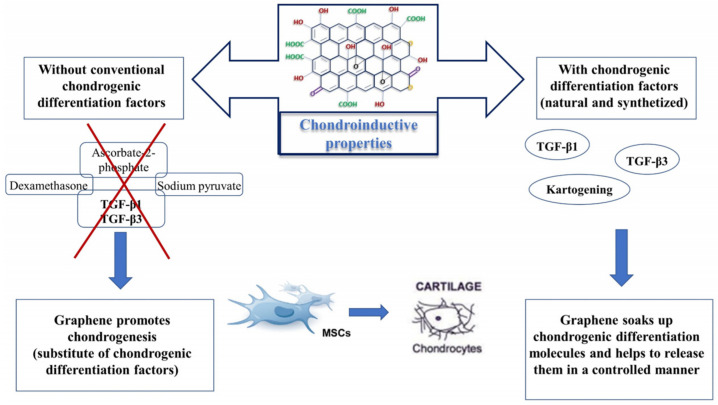
Chondroinductive properties of GO. Graphene oxide promotes chondrogenesis without the addition of conventional chondrogenic differentiation factors in growth media. In parallel, it can enhance chondrogenesis, thus helping to release differentiation factors from biomaterials in a controlled manner. TGF-β1 = transforming growth factor beta 1; TGF-β3 = transforming growth factor beta 3; MSCs = mesenchymal stem cells.

As previously reported for osteogenesis, graphene is a good nanocarrier for several molecules. To induce and improve MSC osteogenesis, researchers typically use a medium supplemented with differentiation or growth factors. Graphene, due to its structure and ability, can soak up molecules dissolved in the medium and improve their entrance into the cells, thereby speeding up the process. The same approach can be applied to induce chondrogenesis in MSCs (Figure 5). With this aim, MSCs can be cultured in the presence of GO derivatives in chondrogenic conditions in the presence of dexamethasone, ascorbate-2-phosphate, sodium pyruvate, and transforming growth factor-beta 1 (TGF-β1) [75]. However, the feasibility of using GO directly as a chondroinductive factor without a differentiation medium has also been investigated. In a recent study, the effect of graphene alone without employing exogenous differentiation factors was explored for the first time [76]. In this study, MSCs were directly cultured in the presence of three different concentrations of GO nanosheets into a photopolymerizable poly(D,L-lactic acid)/polyethylene glycol hydrogel (PDLLA hydrogel). The incorporation of GO into hydrogel did not affect cell viability at all as the GO concentration and chondrogenic markers appeared to be upregulated compared to hydrogels without GO. ECM deposition was also not affected. This study marked the starting point for further evaluations of GO as a chondroinductive material without differentiation medium.

As mentioned earlier, TGF-β1 and TGF-β3 are two of the major chondrogenic differentiation factors and are frequently used alone or in combination with other differentiation molecules in the chondrogenic differentiation medium. During the development of growth plates, BMP signaling promotes the maturation of chondrocytes to facilitate ossification, whereas TGF-beta signaling inhibits hypertrophic differentiation to preserve adequate chondrocytes within the growth plate. Both TGF-beta signaling and BMP signaling are indispensable for the maintenance and repair of articular cartilage [77]. At a molecular level, TGF-β1 improves MSC proliferation and the production of ECM components, thus inducing downregulation of collagen type I expression and upregulation of collagen type II and aggrecan (ACAN) expression. In parallel, TGF-β3 increases production of the ECM components, mainly sulphated glycosaminoglycans (GAG). Their activities are carried out through the Smad pathway, inducing a phosphorylation of Smad that translocates into the MSC nucleus and triggers gene transcription, inducing chondrogenic cell commitment and differentiation [78]. Interestingly, it has been shown that TGF-β activity, particularly of TGF-β3, can induce side-effects, including inflammation, fibroplasia, and a hypertrophic phenotype of mesenchymal stem cells, during chondrogenesis in a time- and concentration-dependent manner [79]. Based on TGF-β’s hypertrophic potential in chondrogenesis, researchers have attempted to induce chondrogenic differentiation using differentiation medium without TGF-β and to improve differentiation using materials such as GO, which can enhance transport and entry of other molecules present in the differentiation medium. A recent study [80] evaluated the ability of poly-Ɛ-caprolactone (PCL) scaffold combined with different concentrations of pristine graphene nanopowders (1%, 3%, 5%, and 10%) to induce chondrogenic MSC differentiation in the presence of TGF-β-free differentiation medium. In vitro cell tests indicated that the prepared grid-like graphene/PCL composite scaffolds possessed good cytocompatibility and nontoxicity for mouse bone marrow MSCs. Stem cells showed good adhesion and proliferation on scaffolds, and they also disclosed a chondrogenic differentiation in the absence of transforming growth factors. In another study, GO was incorporated into alginate-based hydrogels developed for 3D-printed scaffold fabrication. The hydrogel was based on a photocrosslinkable alginate bioconjugated with both gelatin and chondroitin sulfate to mimic the cartilage extracellular matrix, while the nanofiller was based on GO to enhance printability and cell proliferation. The results showed that the incorporation of GO into the hydrogel inks considerably improved the shape fidelity and resolution of 3D-printed scaffolds because of a faster viscosity recovery post extrusion. In vitro assays on human adipose tissue-derived mesenchymal stem cells (hADMSCs) showed that bioconjugated scaffolds presented higher cell proliferation than pure alginate, had good biocompatibility, and induced chondrogenic differentiation without exogenous pro-chondrogenic factors [81].

Even though the extraordinary properties of graphene, in particular GO, can induce chondrogenic differentiation and regeneration without the addition of differentiation factors, graphene can also be used as a nanocarrier for differentiation factors.

### 5.2. Graphene as a Nanocarrier for Natural and Synthetized Chondrogenic Differentiation Factors

Differentiation/growth factors (of natural origin or synthesized) can be administered in situ to obtain rapid repair of local cartilage lesions. However, there are frequent issues to overcome, such as inactivation of these molecules in the site of action or the inability to retain factors at a local level. To overcome these limitations, a possible strategy could be to use carrier materials that are able to release these factors in the interested region. Nevertheless, this approach presents frequent problems, such as the large amount of early release of growth factors from these materials followed by little long-term release [82], which potentially induces side effects, such as TGF-β-related hypertrophic condition. A suitable approach to limit these side-effects is to use a second delivery system that can release growth factors in a controlled manner [83]. With this aim, the use of graphene as a nanocarrier and the use of GO could be a feasible delivery strategy. In a 2014 study [84], GO was used as a nanocarrier to improve controlled TGF-β diffusion into MSCs. Recently, Zhou and coworkers [85] designed a delivery system formed by GO flakes where TGF-β3 was adsorbed and embedded in collagen hydrogels encapsulating MSCs. The effects of this complex compared to exogenously delivered TGF-β3 in culture medium were evaluated with the aim of investigating the capability of GO to adsorb and release active molecules, such as TGF-β3. It was found that GO adsorbed approximately 99% of TGF-β3 molecules and released < 1.7%. Moreover, the gene expression of Sry-type HMG box (SOX9), collagen type II, and ACAN indicated better chondrogenic differentiation of MSCs. Moreover, phosphorylation of Smad2 occurred when TGF-β3 was combined with GO, whereas phosphorylation was strongly reduced in samples with TGF-β3 or GO alone (Figure 5). 

Similarly, rGO can be used as a nanocarrier for specific molecules to improve chondrogenic differentiation. Jiao and coworkers [86] investigated the ability of rGO in combination with gelatin to form a scaffold (rGO–Ge) able to deliver kartogenin (KGN). KGN is a new heterocyclic compound showing high chondrogenic potential [87]. The rGO–Ge + KGN combination promoted MSC chondrogenic differentiation in a synergistic manner. Immunofluorescence and PCR analysis of SOX9, collagen type II, collagen type X, and GAG demonstrated the good pro-chondrogenic effect of this combination. 

These findings suggest GO has good potential in the release of specific molecules, thus improving chondrogenic differentiation, although further investigations are needed to support this hypothesis. In addition, functionalization of the graphene surface with active molecules to enhance the chondrogenic capability of graphene is a new and challenging strategy.

**Table 1:** Biomedical applications of GO. The table summarizes in vitro and in vivo studies related to GO and rGO.

	Graphene Formulation	Biomedical Applications	
De Marco, P. et al. [11]	Collagen membranes enriched with GO	Implementation of bone deposition	In vitro
Radunovic, M. et al. [12]	Collagen membranes enriched with GO	Implementation of bone formation and improvement of the clinical performance of collagen membranes	In vitro
Zarafu, I. et al. [16]	Amines-functionalized GO	Antimicrobial and antibiofilm activity	In vitro
Deng, X. et al. [17]	GO combined with polyethylene glycol (PEG)	Prevention of osteosarcoma invasion	In vitro and in vivo
Di Carlo, R. et al. [19]	GO-coated titanium surfaces	Improvement of properties related to dental implantation materials	In vitro
Jo, S.B. et al. [21]	Polyurethane–nanoGO fibers	Potential matrix for skeletal muscle engineering	In vitro
Bao, D. et al. [22]	Platelet-rich plasma gels with GO (PRP/GO)	Tendon–bone interface healing/supraspinatus tendon reconstruction	In vitro and in vivo
Sadeghianmaryan, A. et al. [23]	Electrospinning polyurethane–GO	Wound dressing	In vitro
Soliman, M. et al. [24]	GO–cellulose nanocomposite	Wound healing	In vitro and in vivo
Llewellyn, S.H. et al. [25]	GO substrates	Peripheral nerve regeneration	In vitro
Dinescu, S. et al. [29]	GO–Chitosan-based 3D scaffolds	Bone tissue engineering	In vitro and in vivo
Son, S.A. et al. [34]	Mesoporous bioactive glass combined with GO quantum dots	Dentin hypersensitivity	In vitro
Yilmaz, E. et al. [37]	HA/GO/COL bioactive composite coating on Ti16Nb	Antibacterial activity,improvement of cell adhesion and viability	In vitro
Kalbacova, M. et al. [38]	Single graphene layer	Improvement of osteoconductivity	In vitro
Nayak, T.R. et al. [39]	Graphene sheets	Acceleration of cell differentiation	In vitro
Arumugam, N. et al. [43]	GO quantum dots	Detection of ascorbic acid	In vitro
Krukiewicz, K. et al. [44]	GO–poly(methyl methacrylate)	Bone tissue engineering	In vitro
Kang, M.S. et al. [45]	rGO–titanium substrates	Dental and orthopaedic bone substitutes	In vitro
Li, Z. et al. [46]	Methacrylated gelatin–GO	Bone tissue engineering	In vitro and in vivo
Kang, E.S. et al. [47]	Gold nanostructure/peptide-nanopatterned GO	Treatment of disorders of bone tissue	In vitro
Zhou, C. et al. [49]	Collagen-functionalized GO	Enhancement of biomimetic mineralization	In vitro and in vivo
Bahrami, S.et al. [50]	rGO-coated collagen scaffolds	Bone tissue engineering	In vitro and in vivo
Fu, C. et al. [51]	L-lysine-functionalized GO nanoparticles on PLGA	Improvement of osseointegration of bone implants	In vitro and in vivo
Kim, J. et al. [52]	Glass slides coated with GO	Upregulation of osteogenic responses	In vitro
Arnold, A.M. et al. [54]	Phosphate–GO releasing inducerons (Ca^2+^ and PO_4_^3−^)	Bone regeneration	In vitro and in vivo
Newby, S.D. et al. [56]	Functionalized graphene nanoparticles	Induction of specific ECM protein expression, bone repair, and regeneration	In vitro
Kim, H.D. et al. [61]	GO incorporated into cryogel-based scaffold	Improvement of osteogenic commitment	In vitro
Di Carlo, R. et al. [65]	GO-decorated cortical membrane	Bone regeneration	In vitro
Di Crescenzo, A. et al. [66]	GO foils	Bone regeneration	In vitro
Bordoni, V. et al. [70]	Monocytes activator GO complexed with calcium phosphate (maGO–CaP)	Immunomodulatory effects in osteogenesis	In vitro and in vivo
Su, J. et al. [71]	GO-coated titanium	Immunomodulatory effects in osteogenesis	In vitro
Chang, T.K. et al. [72]	Graphene and GO particles	Application in orthopaedic prostheses	In vitro and in vivo
Shen, H. et al. [76]	GO-incorporated hydrogel	Biologics-free approach for cartilage tissue engineering	In vitro
Deliormanlı, A.M. et al. [80]	Grid-like graphene/PCL composite scaffolds	Chondrogenic differentiation	In vitro
Olate-Moya, F. et al. [81]	Alginate-based hydrogel with GO	Chondroinductive capability	In vitro
Yoon H.H., et al. [84]	GO sheets	Chondroinductive capability	In vitro
Zhou, M. et al. [85]	Adsorbed TGF-β3 to GO flakes incorporated into collagen hydrogel	Delivering of growth factors and chondrogenic differentiation induction	In vitro
Jiao, D. et al. [83]	Biodegradable gelatin–rGO	Promoting chondrogenic differentiation through kartogenin delivery	In vitro

## 6. Conclusions

Graphene, and in particular its derivative GO, has triggered enormous interest in the scientific and industrial communities owing to its simple synthesis and versatility. Indeed, the graphene chemistry offers a plethora of opportunities for the design of multifunctional nanocomposites and devices with promising uses in tissue engineering. Among its several biomedical applications, GO has been particularly used for bone regeneration purposes as it has been shown to be capable of enhancing and ameliorating osteoconductive properties of materials in vitro and in vivo. Recently, properties of GO have also been successfully applied in the field of cartilage regeneration, achieving promising results in vitro. Very interestingly, graphene-based materials are emerging as attractive materials that are able to modulate inflammatory pathways in immune cells, thus promoting and accelerating tissue regeneration after implantation.

## Data Availability

Data sharing is not applicable for this paper.
